# Implementation and evaluation of a nurse-centered computerized potassium regulation protocol in the intensive care unit - a before and after analysis

**DOI:** 10.1186/1472-6947-10-5

**Published:** 2010-01-25

**Authors:** Miriam Hoekstra, Mathijs Vogelzang, José T Drost, Marcel Janse, Bert G Loef, Iwan CC van der Horst, Felix Zijlstra, Maarten WN Nijsten

**Affiliations:** 1 Department of Anesthesiology, University Medical Center Groningen, University of Groningen, Groningen, the Netherlands; 2 Department of Cardiology, University Medical Center Groningen, University of Groningen, Groningen, the Netherlands; 3 Department of Intensive Care, University Medical Center Groningen, University of Groningen, Groningen, the Netherlands

## Abstract

**Background:**

Potassium disorders can cause major complications and must be avoided in critically ill patients. Regulation of potassium in the intensive care unit (ICU) requires potassium administration with frequent blood potassium measurements and subsequent adjustments of the amount of potassium administrated. The use of a potassium replacement protocol can improve potassium regulation. For safety and efficiency, computerized protocols appear to be superior over paper protocols. The aim of this study was to evaluate if a computerized potassium regulation protocol in the ICU improved potassium regulation.

**Methods:**

In our surgical ICU (12 beds) and cardiothoracic ICU (14 beds) at a tertiary academic center, we implemented a nurse-centered computerized potassium protocol integrated with the pre-existent glucose control program called GRIP (Glucose Regulation in Intensive Care patients). Before implementation of the computerized protocol, potassium replacement was physician-driven. Potassium was delivered continuously either by central venous catheter or by gastric, duodenal or jejunal tube. After every potassium measurement, nurses received a recommendation for the potassium administration rate and the time to the next measurement. In this before-after study we evaluated potassium regulation with GRIP. The attitude of the nursing staff towards potassium regulation with computer support was measured with questionnaires.

**Results:**

The patient cohort consisted of 775 patients before and 1435 after the implementation of computerized potassium control. The number of patients with hypokalemia (<3.5 mmol/L) and hyperkalemia (>5.0 mmol/L) were recorded, as well as the time course of potassium levels after ICU admission. The incidence of hypokalemia and hyperkalemia was calculated. Median potassium-levels were similar in both study periods, but the level of potassium control improved: the incidence of hypokalemia decreased from 2.4% to 1.7% (P < 0.001) and hyperkalemia from 7.4% to 4.8% (P < 0.001). Nurses indicated that they considered computerized potassium control an improvement over previous practice.

**Conclusions:**

Computerized potassium control, integrated with the nurse-centered GRIP program for glucose regulation, is effective and reduces the prevalence of hypo- and hyperkalemia in the ICU compared with physician-driven potassium regulation.

## Background

Hypokalemia and hyperkalemia are both associated with an increased risk of complications that can be potentially fatal [1,2]. Therefore, derangements of blood potassium levels should be avoided in critically ill patients or, when present, rapidly corrected [3-5]. In the intensive care unit (ICU) potassium is administrated continuously by syringe pump, either enterally or parenterally [6-9]. Keeping potassium levels within the normal range (3.5-5.0 mmol/L) requires frequent blood potassium measurements and subsequent adjustments of potassium intake. Although potassium disorders occur frequently in the critical care setting and regulation is considered important, there are only a few studies addressing this subject. Some ICU's use an (nurse-driven) electrolyte replacement protocol [10-12]. However even with this form of standardization, such errors still occur which are an important issue in healthcare systems. For both safety and efficiency, computerized protocols are assumed to be superior over paper protocols [13-19]. In our ICU a nurse-centered computer-assisted glucose regulation program called GRIP (Glucose Regulation in Intensive care Patients) was already fully operational for several years [20,21]. We hypothesized that integration of advice on potassium replacement into this system (GRIP-II) would improve potassium control with no extra or reduced effort of the nurses and physicians. Potassium and glucose regulation share the properties that they both can be measured in a single blood sample on one machine, that both can be delivered by syringe pump, and that both need multiple adjustments per day. Before the implementation of GRIP-II, potassium replacement in our ICU was physician-driven. In this before-after study we describe the extension of GRIP with a potassium intake recommendation algorithm.

## Methods

The before and after study was performed from May 2005 through December 2006 at 2 closed-format, adult ICUs in a 1300 bed tertiary university teaching hospital: a 12-bed surgical ICU and a 14-bed thoracic-surgical ICU. We evaluated potassium regulation with a computerized potassium regulation algorithm that was added to the GRIP program for glucose regulation [20,21]. The primary endpoint was potassium regulation in terms of out of range measurements and speed of correction. The secondary endpoint was endorsement and ease of use by the nurses. At our institution, before implementation of nurse-based computerized potassium regulation, potassium replacement was physician-driven. The physician protocol called for extra potassium infusion when hypokalemia was present. When hyperkalemia developed the potassium administration was stopped. For all patients the physicians explicitly decided each day in the morning what amount of potassium had to be given and entered this amount in the prescription record. Moreover, physicians were frequently consulted by nurses during evening and nights about potassium changes for 30% of the patients. The precise way of executing these guidelines was left to the discretion of the attending physician. This study was approved by the institutional medical ethics review board (METc 2006.185) and informed consent was waived.

### Implementation of a computerized potassium regulation protocol (GRIP-II)

Before the extension of GRIP with a potassium algorithm (GRIP-II, Glucose and potassium Regulation in Intensive care Patients), the GRIP glucose control program was already fully operational at both ICUs at the start of this study. The design and implementation of the freely available nurse-based GRIP program and its glucose control algorithm has previously been described [20]. The GRIP-II potassium protocol was introduced after approval of the medical directors and staff of both intensive care units. No changes were made in the way it was embedded in the routine clinical workflow or its communication with the hospital information system. For the potassium algorithm, we added a limited number of relevant clinical parameters that GRIP-II queries from the hospital information system or the nurse (Table 1).

**Table 1:** Parameters in GRIP and GRIP-II

	From the hospital information system	From the nurse
**Parameters requested for glucose control.**	- Last glucose measurement	- Nurse identification
		- Enteral feeding
		- Stomach retention
		- Intravenous glucose dose
		- Current insulin pump rate
		- Mean arterial pressure > 70 mmHg (yes/no)
		- Noradrenaline dose
		- Dopamin dose
		- Steroid administration (yes/no)
		
**Added parameters for potassium control.**	- Last potassium measurement	- Diuresis over the last 6 hours
	- Last creatinine measurement	- Presence of renal replacement therapy

As the list indicates, four additional input parameters were needed to incorporate potassium control into the existing glucose regulation program. Only two of these parameters had to be entered by nurses.

**Figure 1:**
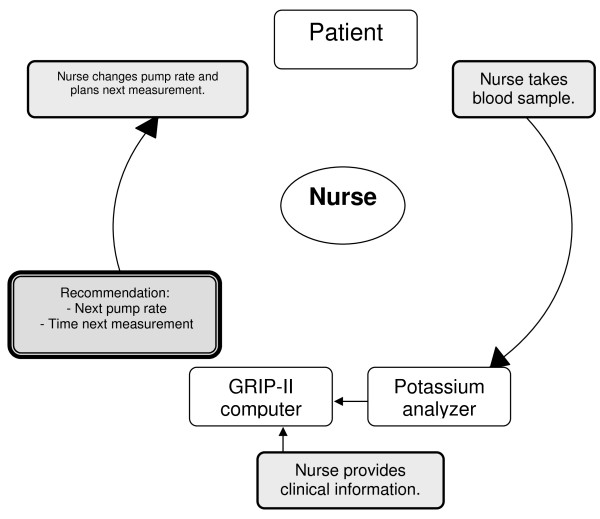
**Nurse-centered potassium regulation cycle with GRIP-II**. Nurse-driven potassium regulation with GRIP-II. After taking a blood sample and analyzing it with the point of care machine, the GRIP-II system automatically retrieves the new potassium value from the hospital information system. GRIP-II then advises about the potassium pump rate, and the time to the next blood sample. This total cycle, including also the glucose measurement and insulin pump rate advice takes 4 minutes and is performed 6 times a day.

After a potassium blood sample was analyzed, GRIP-II provides a recommendation about the desired rate of potassium administration by syringe pump (mmol/hour) and the time interval to the next measurement. In comparison with the previous system, we added the feature to print adhesive labels. For every recommendation, one label is printed that contains the new potassium (and insulin) pump rates. The label is put on the patients' chart. A second label contains both the patients' bar code identification and the time of the next proposed potassium (and/or glucose) measurement. This label is put on a syringe for the next measurement. Thus the risk of incorrect setting of the potassium pump or accidental swapping of samples is minimized [22]. As with glucose, the drawing of blood, the measurement of the potassium level, getting the advice from GRIP-II and changing the potassium pump was one coherent action for nurses (Figure 1).

### Architecture of the potassium algorithm

**Figure 2:**
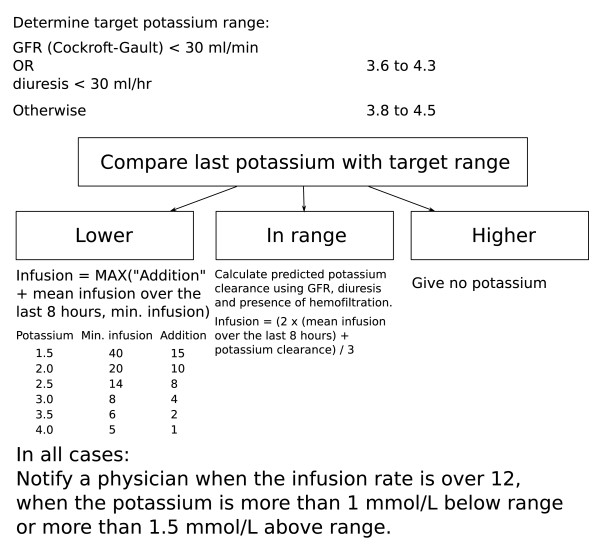
**Diagram of the GRIP-II potassium algorithm**. A schematic diagram of the potassium infusion recommendation algorithm of GRIP-II. Note that the exact source code to the algorithm is freely downloadable from the project web site http://grip-glucose.sf.net/.

The potassium target before the implementation of the computerized decision support system was in the middle of the normal range (reference values 3.5-5.0 mmol/L), i.e. between around 4.0 and 4.5 mmol/L. We designed the potassium control algorithm to aim for similar potassium levels and defined the preferred range of potassium values as 3.8 to 4.5. In the case of impaired renal function (estimated creatinine clearance <30 ml/min using the Cockroft-Gault formula [23] or diuresis of less than 30 ml/hour) GRIP-II lowered the target range for such patients by 0.2 mmol/L. When making a decision, the algorithm first categorized the situation into three possible states: hypokalemia (potassium < target range), normokalemia (potassium within the target range) and hyperkalemia (potassium > target range). When the potassium was categorized as too low (<3.8 mmol/L), potassium infusion was advised to be started following a sliding scale algorithm. Starting values ranged from 20 mmol/h for a potassium level of 2.0 decreasing to 6 mmol/h for a potassium level of 3.5 mmol/L. When the potassium was categorized as within-range, the algorithm calculated the mean potassium infusion over the last 8 hours, or since admission, whichever was shorter, and advised this rate (provided a normal potassium clearance, see below). When the potassium was categorized as too high (>4.5 mmol/L), the algorithm advised no potassium infusion. In case of a distinctly abnormal potassium value (i.e. potassium <2.8 or potassium >6.0 mmol/L or in case of renal failure <2.6 or >5.8 mmol/L), GRIP-II requested prompt notification of the attending physician in addition to providing its pump rate advice and next measurement advice. In case of a significant or symptomatic hyperkalemia the patient was treated according general guidelines by the physician. GRIP-II does not provide advice about those interventions. An overview of the GRIP-II potassium algorithm is given in figure 2. The potassium clearance is calculated as the fraction of a normal person's clearance. The value is by default 1, and is lowered for a glomerular filtration rate (GFR) <100, or for diuresis <60 ml/hr, in a linear fashion (i.e., a diuresis of 6 ml/hr would translate to a clearance of 0.1). When the patient is on hemofiltration, 0.5 is added to the clearance. The translation of clearance to pump rate is by multiplying by 4 (so a clearance of 1 would make the pump converge to 4, a clearance of 0.25 to 1, etc.). The recommendations generated by GRIP-II with regard to the advised dose or the advised measurement interval could be overruled or adjusted by nurses or physicians at any time; such instances were automatically recorded. As we identified no contra-indications for using the GRIP-II system, all admitted patients during the study period were managed by GRIP-II within 1 hour after arrival at the ICU. When a patient had recovered enough to take his own meals and thus was also unsuitable for continuous insulin infusions, he or she was taken out of GRIP-II altogether.

### Potassium measurements

Potassium (and glucose) levels were determined in lithium-heparin anticoagulated blood in 1.5 ml syringes (Pico, Radiometer Copenhagen) taken from an arterial line [24]. Potassium level was determined with an ion-selective method by a point of care (POC) blood gas analyzer (ABL-Radiometer 700 or 800 series) present at the ICU. The minimal sample size needed was 0.1 ml. The POC-analyzer submitted measurements to the central hospital information system, from which measurements were queried by GRIP-II. Serum potassium measurements were only rarely used, as measurements in anticoagulated blood should not be directly compared to serum measurements, since serum potassium is sensitive to the phenomenon of pseudohyperkalemia [25]. Hypokalemia was defined as a potassium level <3.5 mmol/L and hyperkalemia as a potassium level >5.0 mmol/L.

### Potassium administration

Patients received potassium either enterally or by a central venous catheter. Before implementation of GRIP-II the potassium intake was reviewed daily by the attending physician or more frequently in case of marked hypo- or hyperkalemia. To minimize errors in (the interpretation of) dosing potassium was administered in a "one-to-one" solution (50 mmol of potassium chloride in 50 mL) by a syringe pump either to a central venous catheter or to a gastric, duodenal or jejunal tube. As with insulin, only continuous delivery without boluses was used for potassium administration. In all cardiac surgery patients, magnesium suppletion of 30 mmol/day was given and in other patients in case of hypomagnesemia (i.e. < 0.80 mmol/L) [26].

### Data collection and analysis

At the moment of data collection, the GRIP program had been running for 16 months at the surgical ICU and for 11 months at the cardiothoracic ICU. We included patients who were admitted between 6 months before and 12.5 months after implementation of GRIP-II at the surgical ICU and between 6 months before and 10.5 months after implementation of GRIP-II at the thoracic ICU. All patients admitted during those study periods were included. Information was collected both from the GRIP-II system itself and from the central hospital information system. Actual compliance of the nurses with the recommendations given by the program was recorded. Any change from the advice of GRIP-II was counted as non-compliance. With written inquiries we measured the impact of the computer program as perceived by the nurses and their opinion about (computerized) potassium control six months after the program had become part of routine care.

Before further analysis we calculated each patient's potassium curve using linear interpolation. With these individual curves, the prevalence of hypokalemia (or hyperkalemia) was calculated as the duration in the hypokalemic range (or hyperkalemic range) divided by the total duration of ICU-stay [27]. Means were expressed with a standard deviation (SD) and compared with the Students t-test. In the case of skewed data (as assessed with kurtosis) medians with interquartile ranges (IQR) were used, and compared with the Mann-Whitney U test. Categorical variables were compared with the Chi-square test. A double-sided P value < 0.05 was considered significant. All statistical analysis was performed using SPSS (Statistical Package for the Social Sciences) version 15.0.

## Results

We studied 2210 patients (775 before and 1435 after implementation of GRIP-II) over a period of 14917 patient-ICU days. Table 2 shows the patient characteristics of both groups. Altogether, the mean age was 62 ± 15 years and 1452 patients (66%) were male. In both groups cardiothoracic surgery was the main reason of admission (41% before, and 43% after implementation). There were no baseline differences between the two groups with the exception of baseline creatinine, but with no difference in the development of acute kidney injury or the need for renal replacement therapy during ICU admission. A total of 53574 potassium measurements were analyzed, of which 15857 were before and 37717 after the introduction of computer-assisted potassium regulation. The majority of these measurements were combined potassium/glucose measurements. The total number of potassium/glucose measurements rose from 5.3 to 5.9 per patient-day (P < 0.001).

**Table 2:** Comparison of patient groups before and after the implementation of computerized potassium regulation with GRIP-II.

	Before GRIP-II	After GRIP-II	P-value
N	775	1435	
Age (mean ± SD)	62 ± 16	61 ± 15	ns
Male sex (%)	67	65	ns
Reason of admission (%)			
Abdominal surgery	13.5	12.3	
Cardiac	16.8	18.1	
Medical	7.4	6.8	
Miscellaneous	4.8	4.3	
Neurological	1.3	2.9	
Oncologic	2.3	2.1	
Cardiothoracic surgery	40.8	43.2	
Trauma	7.5	5.5	
Vascular surgery	5.7	4.9	
APACHE II (mean ± SD) *	15 ± 7	14 ± 7	ns
Admission creatinine level, μmol/L (mean ± SD)	91 ± 56	98 ± 78	0.035
Acute kidney injury during ICU admission (%)^†^	10.2	11.8	ns
Renal replacement therapy (%)^+^	5.6	4.1	ns
Point of Care potassium/glucose measurements (patient-day)^-1^	5.3	5.9	<0.001
Length of stay (days) at the ICU			
Cardiothoracic ICU	1.0 (0.9-3.1)	1.0 (0.9-2.6)	ns
Surgical ICU	3.5 (1.3-8.8)	3.6 (1.1-9.7)	ns
Length of hospital stay (days)			
Cardiothoracic ICU	12 (8-22)	11 (8-19)	ns
Surgical ICU	19 (12-36)	19 (11-41)	ns
Hospital mortality (%)			
Cardiothoracic ICU	8	7	ns
Surgical ICU	13	15	ns

*Only for surgical patients

† Acute kidney injury defined as a two-fold increase in creatinine levels.

+ Renal replacement therapy during the first 5 days of ICU-admission

Characteristics of the two patient groups. Numbers are expressed as percentages or median (IQR) unless otherwise specified. Abbreviations used in table: GRIP-II, Glucose and potassium Regulation in Intensive care Patients; SD, Standard Deviation; ns, not significant; APACHE II, Acute Physiology And Chronic Health Evaluation II score; ICU, Intensive Care Unit.

**Figure 3:**
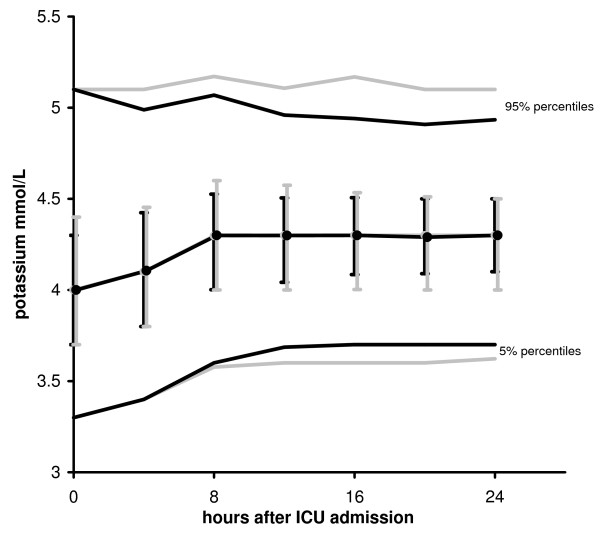
**Time course of potassium**. Time course of medians with 25- and 75-percentiles (i.e. interquartile ranges, indicated by error bars) and 5- and 95-percentiles of potassium during the first ICU day. The grey lines reflect the situation before and the black lines reflect the situation after GRIP-II. With preservation of the same median potassium levels, GRIP-II achieved a lower number of potassium levels that were out of range (P < 0.001).

Under GRIP-II, 92% of all patients received potassium infusion during their stay at the ICU. The median (IQR) fraction of time on potassium infusion was 87% (62-97%) of total ICU length of stay. Mean ± SD potassium administration rate was 2.3 ± 1.5 mmol/hour. Figure 3 shows the change in plasma potassium level during the first ICU day before and after the introduction of GRIP-II. In both groups the median potassium levels moderately increased from 4.0 at admission to 4.3 mmol/L 8 h after ICU-admission. The median levels or their time course during the first 24 hours did not change but the variation of these values from 24 hours after admission onward decreased significantly after the introduction of GRIP-II (P < 0.001). When all potassium levels measured after the first 8 h of ICU admission were analyzed, the incidence of hypokalemia decreased from 2.4% to 1.7% (Odds ratio [OR] 1.4; 95% confidence interval [CI] 1.3-1.6; P < 0.001) and hyperkalemia from 7.4% to 4.8% (OR 1.6; 95% CI 1.5-1.7; P < 0.001). The prevalence of hypokalemia decreased from 2.4% to 1.9% (OR 1.3; CI 1.2-1.4 P < 0.001) and the prevalence of hyperkalemia decreased from 6.2% to 4.3% (OR 1.4; CI 1.4-1.5; P < 0.001). This reduction was more pronounced for the extreme values in the hyperkalemic range as the prevalence of marked hyperkalemia (>6.0 mmol/L) decreased from 2.2% to 1.0% (OR 2.3; CI 2.2-2.5; P < 0.001). In 14 patients who presented with a hypokalemia of 3.0 mmol/L or less (median: 2.9 mmol/L, IQR: 2.7 - 3.0 mmol/L), target range was achieved in a median time of 4 hours. The formal involvement of physicians in potassium regulation decreased from 1.3 times per patient per day to 0.04 times per patient per day.

Questionnaires were anonymously filled out by 76 of the 125 ICU nurses (Table 3). The majority considered that potassium regulation with GRIP-II an improvement compared to the old physician-driven potassium regulation. They experienced GRIP-II as easy to work with and reliable. Also the labelling system was considered effective. Nurses exactly followed the recommended pump rates 18% of time in the first week of implementation. In subsequent weeks, compliance rates rapidly rose to 19%, 49%, 91%. Beyond 5 weeks, potassium administration was fully regulated by GRIP-II (without interference of a physician or overruled recommendations) in >95% of the time.

**Table 3:** Nurses' opinion on computerized potassium control (GRIP-II).

Question	Totally disagree	Disagree	Neutral	Agree	Totally agree
Potassium control is important.	0	0	1	33	65
Working with GRIP-II is simple.	3	3	3	32	61
GRIP-II is a good tool to regulate potassium with.	5	4	8	50	33
GRIP-II is an improvement over the previous potassium control.	4	7	25	30	34
GRIP-II is reliable.	1	9	28	51	11
You feel more secure about preventing hyperkalemia with GRIP-II.	12	13	30	33	12
The labelling system is effective	5	3	12	49	31
Less frequent consultation of the attending physician about potassium after the introduction of GRIP-II is an advantage.	3	0	11	33	54

Results of the nurse questionnaires regarding their opinion on potassium control with GRIP-II compared with conventional potassium control. A total of 125 questionnaires were distributed with a response rate of 61%. The numbers are percentages. The total percentages do not always add up to 100 since a few nurses were unable to choose one of the predefined answers. As the answers indicate, the majority of the nurses consider GRIP-II to be an improvement.

## Discussion

Incorporation of a potassium replacement algorithm into an already existing computerized clinical decision support system for glucose regulation led to a reduced prevalence of abnormal potassium levels compared with physician-driven potassium regulation in the ICU.

The relevance of avoiding severe potassium abnormalities cannot be overstated. In the ICU both hypokalemia and hyperkalemia frequently occur. Disorders in the potassium homeostasis are associated with serious complications like cardiac arrhythmias and sudden death [1]. Many cases of abnormal potassium values are iatrogenic and probably sometimes even the result of frank errors [10-12]. We developed and implemented a nurse-driven computerized potassium regulation protocol to improve efficiency of potassium regulation and patient safety. The GRIP-II program was specifically designed to reduce the potential errors. Example of a safety feature, the labelling system, which was considered by the nurses to be effective, was introduced to eliminate written copied instructions. Also in case of a markedly abnormal potassium level (<2.8 mmol/L or >6.0 mmol/L) the attending physician must be notified, so that GRIP-II in fact is also a computerized alert system. Likewise, GRIP-II requests physician notification in case of potassium infusion rates over 12 mmol/h. It should be noted that the improved potassium regulation in both directions (i.e. in preventing both hypokalemia and hyperkalemia) was achieved only with regulating potassium infusion. GRIP-II does not provide other advices on hyperkalemia apart from stopping potassium infusion.

We believe that the care taken to integrate GRIP-II into the nurses' workflow has contributed to its success, since this is known to be essential to make a clinical decision support system a success [28]. As described, the entire routine (from taking a blood sample to changing the potassium and insulin infusion rates) is one coherent action that takes about 4 minutes. The addition of potassium regulation to the GRIP program did not add any significant nursing time or costs, because computerized glucose control was already routine practice and potassium and glucose are measured from the same blood sample, and only two additional parameters had to be entered into the GRIP-II computer. Our data show, that after only 5 weeks more than 95% of the time all potassium levels were completely regulated by GRIP-II. Our nurses were already used to working with the GRIP program for glucose control, so when implemented in other institutions compliances rates will probably increase more gradually.

Remarkably, very few papers have explicitly described strategies how to regulate potassium in ICU patients [8-12,17,18,29]. Analogous to glucose control, potassium control may even be perceived as tedious. When physicians must be involved in all minor derangements of an ICU patient, the necessary (telephonic) consultations with the attending physician may be viewed as neither particularly efficient nor optimal as physicians caring for ICU patients have more than sufficient inherently complex problems to handle, many of which cannot be handled by algorithms such as GRIP. As with glucose-control, it has been demonstrated that protocol-driven electrolyte replacement is superior to physician-driven electrolyte replacement in the intensive care setting [10-12]. A prospective study by Hijazi et al comparing protocol-driven and physician-driven potassium, magnesium and phosphate replacement concluded that protocol-driven replacement was associated with more efficient regulation (timely replacement and fewer missed low levels) [12]. In a small retrospective study the implementation of an electrolyte dosing order form resulted in greater efficiency for potassium replacement without an increase in the number of adverse events [10]. However, the study group was too small to draw conclusions about safety. Kanji et al evaluated the implementation of a paper electrolyte replacement protocol in 63 ICU patients and also this protocol resulted in more efficient electrolyte replacement [11]. To our knowledge, there are no reports of a computerized potassium recommendation protocol in the ICU. Computer software is recognized as a tool to reduce serious medication errors and improve adherence to recommended care [13,14,17,30].

### Limitations

Our study has a number of limitations. Although the importance of avoiding frank hypokalemia or hyperkalemia is obvious, especially in critically ill patients, it is not known if 'strict' potassium control, like glucose control, would be of clinical benefit [31]. Also it is not known if computerized decision support systems improve patient outcomes [32]. Our study was not designed or powered to detect differences in the outcome of ICU patients who received the computerized potassium control achieved by GRIP-II. Response of the nurses to the questionnaire was not complete but only 61%.

Because of the before-after study design, potential bias could have been introduced in to our study. Patients were not randomly assigned to physician-driven or computer-driven potassium control. Between the two study groups, there were a significantly higher number of potassium measurements per patient per day. We believe that the rise in the average number of requested blood samples from 5.3 to 5.9 per day was not caused by the addition of the potassium algorithm but by the sharpened glucose control during the study period as most samples were combined potassium and glucose measurements. Another limitation is that before the implementation of a computerized protocol, potassium control was physician-driven. Conceivably the introduction of a nurse-based paper-protocol would also have improved potassium control. However, computerized protocols are known to have several advantages over paper-protocols, such as better compliance and more efficient control [14].

### Future perspectives

Analogous to studies on intensive insulin therapy, relations between potassium and outcome can only be investigated when an adequate protocol and infrastructure for realizing potassium control are in place [33]. We believe this can be achieved with systems such as the GRIP-II program. Likewise interactions between glucose, insulin and potassium as they are presumed to occur with the administration of GIK (glucose-insulin-potassium) in acute myocardial infarction, can better be studied with a system such as GRIP-II [34].

Several variables that influence the potassium regulation are not considered in our potassium regulation protocol. For example, the effect of acid-base disorders, insulin infusion and potassium administration route are possible extensions of the GRIP-II potassium algorithm. We aim to combine the potassium and the glucose algorithm in the future, so that for example GRIP-II can anticipate that a high insulin infusion can decrease potassium levels and thus increase the potassium infusion at the same time. The advantage of a computerized clinical decision support system over a paper-protocol is that it can employ a complex algorithm, while maintaining a simple interface.

Our study concerned the extensions of a computer decision support system specific to our ICU. Although the code of the system is freely available on the internet, to our knowledge no other ICUs have implemented it thus far. Successful introduction will also depend on a structured educational effort. Unlike most computerized decision support systems, GRIP-II is independent of a patient data management system (PDMS) which makes it widely applicable. Likewise, if an institution prefers diluted potassium chloride over a concentrated solution, the recommendation concerning the infusion rate of GRIP can be easily converted. We think the usefulness of the integration of potassium with insulin recommendation is not limited to our system. The algorithm behind potassium control is relatively simple, as outlined in the methods section. This could easily be added to a paper insulin protocol or different computer system than ours.

## Conclusion

In conclusion, computerized potassium control, integrated with a nurse-centered program for glucose regulation (GRIP) is safe, effective and reduces the prevalence of hypo- and hyperkalemia in the ICU.

## Note

The program code for the GRIP-II program is freely available as open source from http://grip-glucose.sf.net/.

## Abbreviations

ICU: intensive care unit; GRIP: glucose regulation in intensive care patients; GRIP-II: glucose and potassium regulation in intensive care patients; GFR: glomerular filtration rate; POC: point-of-care; SD: standard deviation; IQR: interquartile range; SPSS: statistical package for the social sciences; OR odds ratio; CI: confidence interval; GIK: glucose-insulin-potassium; PDMS: patient data management system; APACHE II: acute physiology and chronic health evaluation II score.

## Competing interests

The authors declare that they have no competing interests.

## Authors' contributions

MH, MV, FZ and MN participated in study design. MH, MV, JD, MJ and MN participated in data acquisition and analysis. MV and MN participated in programming the computer software. MH and MV participated in drafting and interpretation of the manuscript. MN, FZ, IvdH and BL participated in interpretation of the manuscript. All authors read and approved the final manuscript.

## Pre-publication history

The pre-publication history for this paper can be accessed here:

http://www.biomedcentral.com/1472-6947/10/5/prepub

## References

[B1] Rose BD Post TW Clinical Physiology of Acid-Base and Electrolyte Disorders 2001 New York, McGraw-Hill

[B2] Halperin ML Kamel KS Potassium Lancet 1998 352 135 140 9672294 10.1016/S0140-6736(98)85044-7

[B3] Weisberg LS Szerlip HM Cox M Disorders of potassium homeostasis in critically ill patients Crit Care Clin 1987 3 835 854 3332226

[B4] Weisberg LS Management of severe hyperkalemia Crit Care Med 2008 36 3246 3251 10.1097/CCM.0b013e31818f222b 18936701

[B5] Gennari FJ Disorders of potassium homeostasis. Hypokalemia and hyperkalemia Crit Care Clin 2002 18 273 288 10.1016/S0749-0704(01)00009-4 12053834

[B6] Hamill RJ Robinson LM Wexler HR Moote C Efficacy and safety of potassium infusion therapy in hypokalemic critically ill patients Crit Care Med 1991 19 694 699 10.1097/00003246-199105000-00016 2026032

[B7] Kruse JA Clark VL Carlson RW Geheb MA Concentrated potassium chloride infusions in critically ill patients with hypokalemia J Clin Pharmacol 1994 34 1077 1082 7876399 10.1002/j.1552-4604.1994.tb01984.x

[B8] Kraft MD Btaiche IF Sacks GS Kudsk KA Treatment of electrolyte disorders in adult patients in the intensive care unit Am J Health-Syst Pharm 2005 62 1663 1682 10.2146/ajhp040300 16085929

[B9] Sedlacek M Schoolwerth AC Remillard BD Electrolyte disturbances in the intensive care unit Semin Dial 2006 19 496 501 10.1111/j.1525-139X.2006.00212.x 17150050

[B10] Owen P Monahan MF MacLaren R Implementation and assessing an evidence-based electrolyte dosing order form in the medical ICU Intensive Crit Care Nurs 2008 24 8 14 10.1016/j.iccn.2007.04.006 17686630

[B11] Kanji Z Jung K Evaluation of an electrolyte replacement protocol in an adult intensive care unit: a retrospective before and after analysis Intensive Crit Care Nurs 2009 25 181 189 10.1016/j.iccn.2009.03.004 19398203

[B12] Hijazi M Al-Ansari M Protocol-driven vs. physician-driven electrolyte replacement in adult critically ill patients Ann Saudi Med 2005 25 105 110 15977686 10.5144/0256-4947.2005.105 PMC6147962

[B13] Kaushal R Shojania KG bates DW Effects of computerized physician order entry and clinical decision support systems on medication safety: a systematic review Arch Intern Med 2003 163 1409 1416 10.1001/archinte.163.12.1409 12824090

[B14] Hunt DL Haynes RB Hanna SE Smith K Effects of computer-based clinical decision support systems on physician performance and patient outcomes: a systematic review JAMA 1998 280 1339 1346 10.1001/jama.280.15.1339 9794315

[B15] Tierney WM Improving clinical decisions and outcomes with information: a review Int J Med Inf 2001 62 1 9 10.1016/S1386-5056(01)00127-7 11340002

[B16] Boord JB Sharifi M Greevy RA Griffin MR Lee VK Webb TA May ME Waitman LR May AK Miller RA Computer-based insulin infusion protocol improves glycaemic control over manual protocol J Am Med Inform Assoc 2007 14 278 287 10.1197/jamia.M2292 17329722 PMC2244871

[B17] Hemstreet BA Stolpman N Badesch DB May SK McCollum M Potassium and phosphorus repletion in hospitalized patients: implications for clinical practice and the potential use of healthcare information technology to improve prescribing and patient safety Curr Med Res Opin 2006 22 2449 2455 10.1185/030079906X148463 17257459

[B18] Paltiel O Gordon L Berg D Isreali A Effect of a computerized alert on the management of hypokalemia in hospitalized patients Arch Intern Med 2003 163 200 204 10.1001/archinte.163.2.200 12546610

[B19] Hoekstra M Vogelzang M Verbitskiy Nijsten MW Health technology assessment review: Computerized glucose regulation in the intensive care unit - how to create artificial control Crit Care 2009 13 223 10.1186/cc8023 19849827 PMC2784347

[B20] Vogelzang M Zijlstra F Nijsten MW Design and implementation of GRIP: a computerized glucose control system at a surgical intensive care unit BMC Med Inform Decis Mak 2005 5 38 10.1186/1472-6947-5-38 16359559 PMC1334184

[B21] Vogelzang M Loef BG Regtien JG Horst ICC van der van Assen H Zijlstra F Nijsten MWN Computer-assisted glucose control in critically ill patients Intensive Care Med 2008 34 1421 1427 10.1007/s00134-008-1091-y 18389221 PMC2491417

[B22] Horsky J Kuperman GJ Patel VL Comprehensive analysis of a medication dosing error related to CPOE J Am Med Inform Assoc 2005 12 377 382 10.1197/jamia.M1740 15802485 PMC1174881

[B23] Gault MH Longerich LL Harnett JD Wesolowski C Predicting glomerular function from adjusted serum creatinine Nephron 1992 62 249 256 10.1159/000187054 1436333

[B24] José RJ Preller J Near-patient testing of potassium levels using arterial blood gas analysers: can we thrust these results? Emerg Med J 2008 25 510 513 10.1136/emj.2007.053322 18660404

[B25] Nijsten MW deSmet BJ Dofferhoff AS Pseudohyperkalemia and platelet counts N Engl J Med 1999 325 1107 10.1056/NEJM199110103251515 1891016

[B26] Hamill-Ruth RJ McGory R Magnesium repletion and its effect on potassium homeostasis in critically ill adults: results of a double-blind, randomized, controlled trial Crit Care Med 1996 24 38 45 10.1097/00003246-199601000-00009 8565536

[B27] Vogelzang M Horst IC van der Nijsten MW Hyperglycemic index as a tool to assess glucose control: a retrospective study Crit Care 2004 3 R122 127 10.1186/cc2840 PMC468891 15153239

[B28] Kawamoto K Houlihan CA Balas EA Lobach DF Improving clinical practice using clinical decision support systems: a systematic review of trials to identify features critical to success BMJ 2005 330 765 10.1136/bmj.38398.500764.8F 15767266 PMC555881

[B29] Cohn JN Kowey PR Whelton PK Prisant LM New guidelines for potassium replacement in practice: a contemporary review by the national council on potassium in clinical practice Arch Intern Med 2000 160 2429 2436 10.1001/archinte.160.16.2429 10979053

[B30] Garg AX Adhikari NKJ McDonald H Rosas-Arellano MP Devereaux PJ Beyene J Sam J Haynes RB Effects of computerized clinical decision support systems on practitioner performance and patient outcomes: a systematic review JAMA 2005 293 1223 1238 10.1001/jama.293.10.1223 15755945

[B31] Berghe G van den Wouters P Weekers F Verwaest C Bruyninckx F Schetz M Vlasselaers D Ferdinande P Lauwers P Bouillon R Intensive insulin therapy in critically ill patients N Engl J Med 2001 345 1359 1367 10.1056/NEJMoa011300 11794168

[B32] Mollon B Chong JJR Holbrook AM Sung M Thabane L Foster G Features predicting the success of computerized decision support for prescribing: a systematic review of randomized controlled trials BMC Med Inform Decis Mak 2009 9 11 10.1186/1472-6947-9-11 19210782 PMC2667396

[B33] He FJ MacGregor GA Fortnightly review: Beneficial effects of potassium BMJ 2001 323 497 501 10.1136/bmj.323.7311.497 11532846 PMC1121081

[B34] Puskarich MA Runyon MS Trzeciak S Kline JA Jones AE Effect of Glucose-Insulin-Potassium Infusion on Mortality in Critical Care Settings: A Systematic Review and Meta-Analysis J Clin Pharmacol 2009 49 758 767 10.1177/0091270009334375 19417124 PMC2918881

